# Commencement Exercises of the Atlanta College of Physicians and Surgeons

**Published:** 1908-06

**Authors:** 


### COMMENCEMENT EXERCISES OF THE ATLANTA COLLEGE OF PHYSICIANS AND SURGEONS.

The Atlanta College of Physicians and Surgeons awarded diplomas to thirty-three physicians and fifty-five dentists, April 24.

The report of Dr. W. S. Elkin, dean of the medical department, showed that during the year just closed, there were 224 students in the medical department, 180 in the dental department and 121 in the pharmaceutical department.

Certificates of proficiency were presented to the following young physicians:

Dr. G. L. Bush, of Georgia; Dr. T. C. Hodge, of Georgia; Dr. Shields, of Georgia, and Dr. N. J. Newman, of Georgia.

The graduates were:

J. O. Baldwin, J. H. Baxter, W. L. Beauchamp, L. H. Bishop, B. S. Branham, G. L. Bush, J. L. Cheshire, G. L. Echols, D. B. Edwards, W. E. Fulmer, J. A. Green, G. O. Gunter, T. C Hodge, W. C. Howell, C. T. Key, F. V. Meriwether, S. R. Methvin, W. C. Miles, S. E. McCotter, N. J. Newson, A. R. Parrott, J. E. Pressly, J. L. Shepard, H. F. Shields, J. T. Smith, R. J. Spier, R. M. Stephenson, C. C. Stockard, Jr., and L. R. Weeks.

### PURE MILK, CREAM AND ICE CREAM.

The Atlanta Board of Health is now preparing to enforce an ordinance recently passed and undoubtedly is a wise one and should receive the hearty support of the medical profession. The law provides that:

"All vehicles used for hauling or distributing milk or cream must be kept neat and clean, and in good repair, and must not be used for hauling manure, slops or anything else of an objectionable nature, and must be provided with a covered top of canvass or other material which will protect all vessels containing milk of cream from the rays of the sun.

"Cream sold, or offered, or kept for sale as such must contain at least 20 per cent butter fats, and must not contain any foreign substances or coloring matter, and must not contain more than 500,000 bacteria per cubic centi-meter.

"Ice cream sold or kept for sale must contain at least 10 per cent. butter fats for fruit ice cream, and 12 per cent. for plain ice cream."

